# Afatinib and radiotherapy, with or without temozolomide, in patients with newly diagnosed glioblastoma: results of a phase I trial

**DOI:** 10.1007/s11060-021-03877-6

**Published:** 2021-11-17

**Authors:** Frank Saran, Liam Welsh, Allan James, Catherine McBain, Rao Gattamaneni, Sarah Jefferies, Fiona Harris, Karine Pemberton, Jennifer Schaible, Shaun Bender, Agnieszka Cseh, Michael Brada

**Affiliations:** 1 The Royal Marsden NHS Foundation Trust, London, UK; 2 Present Address: Cancer and Blood Service, Auckland City Hospital, Building 8, 99 Park Road, Grafton, Private Bag 92024, Auckland, 1142 New Zealand; 3 The Beatson West of Scotland Cancer Centre, Glasgow, UK; 4 The Christie NHS Foundation Trust, Manchester, UK; 5 Cambridge University Hospitals NHS Foundation Trust, Cambridge, UK; 6 Boehringer Ingelheim Ltd, Bracknell, UK; 7 Boehringer Ingelheim Pharma GmbH & Co. KG, Biberach, Germany; 8 Boehringer Ingelheim Pharmaceuticals, Inc., Ridgefield, CT USA; 9 Boehringer Ingelheim International GmbH, Ingelheim, Germany; 10 Clatterbridge Cancer Centre NHS Foundation Trust, Bebington, UK

**Keywords:** Glioblastoma, Afatinib, Dose-escalation, Temozolomide, Radiotherapy

## Abstract

**Background:**

Glioblastoma multiforme (GBM) is the most common primary malignant brain tumor in adults. Amplification or overexpression of the epidermal growth factor receptor gene, part of the ErbB family, occur in approximately 40% and 60% of patients with GBM, respectively. We present data from a dose-finding study of the ErbB inhibitor afatinib in combination with radiotherapy (RT), with or without temozolomide (TMZ), in patients with GBM.

**Methods:**

This was a phase I, open-label, 3 + 3 dose-escalation trial in patients with newly-diagnosed, histologically-confirmed grade 4 malignant glioma and proven O^6^-methylguanine-DNA methyltransferase gene promoter methylation status. The primary endpoint was the maximum tolerated dose (MTD) of continuous daily afatinib when given in combination with RT, with (regimen M) or without (regimen U) concomitant TMZ treatment.

**Results:**

Fifty-five patients were enrolled; 36 received ≥ 1 dose of trial medication (regimen M, n = 20, regimen U, n = 16). Afatinib was discontinued by all patients during the study. Reasons for afatinib discontinuation (regimen M/U) included disease progression (45%/50%), dose-limiting toxicity (10%/0%), and other adverse events (AEs; 35%/38%). The most frequently reported AEs with either regimen were diarrhea and rash, with no new safety signals identified. The MTD was determined as afatinib 30 mg in combination with daily TMZ and RT, and afatinib 40 mg in combination with RT alone.

**Conclusions:**

This study identified the MTD for afatinib in combination with RT, with and without TMZ, in patients with GBM. Further studies of afatinib in patients with GBM are warranted and should be based on appropriate biomarker-based preselection.

**Trial registration:**

NCT00977431 (first posted September 15, 2009).

**Supplementary Information:**

The online version contains supplementary material available at 10.1007/s11060-021-03877-6.

## Introduction

Glioblastoma multiforme (GBM) is the most common malignant primary brain tumor in adults [1] and is associated with a poor prognosis, with a median progression-free survival (PFS) of 7.4‒10.7 months [2–4] and median overall survival (OS) of 14.6 months [5]. Limited progress has been made in improving outcomes for patients with GBM in recent decades [1]. First-line therapy for newly diagnosed patients is maximal safe surgical resection, followed by radiotherapy (RT) and temozolomide (TMZ) [6]. However, response to TMZ treatment can vary depending upon the methylation status of the methyl-guanine methyl transferase (*MGMT*) enzyme promoter [7, 8]. Additionally, most glioblastomas become resistant to first-line therapies, which can occur via several mechanisms, including activation of DNA repair mechanisms, evasion of apoptosis, and adaptation of the cell cycle [9, 10]. As survival rates remain low, there is a large unmet need in GBM, particularly for patients with unmethylated *MGMT* promoters, for whom standard treatments are less effective [1, 11]. Consequently, several biomarker-driven therapeutic targets have been investigated to date, including the ErbB family of receptors.

Dysregulation of the ErbB pathway has been reported to contribute to GBM progression [12], with mutation, rearrangement, altered splicing and/or focal amplification of the epidermal growth factor receptor (*EGFR*) gene observed in over half of GBM cases [13–15]. Some studies have indicated that overexpression of EGFR may be associated with worse outcomes following RT in patients with GBM [16, 17]. EGFR tyrosine kinase inhibitors (TKI) have therefore been investigated in patients with malignant glioma or GBM, but have so far shown little activity in this setting [18]. Afatinib is an ErbB-family blocker that is approved for use in patients with NSCLC [19, 20]; it irreversibly binds to and blocks EGFR (ErbB1), HER2 (ErbB2), and ErbB4. Afatinib is therefore considered to have a wider inhibitory profile than first-generation EGFR TKIs [21, 22]. Furthermore, brain penetrance is recognized as a potential hurdle in the utilization of EGFR TKIs in GBM [23, 24]. Preclinical data indicate that afatinib has a moderate capacity to penetrate the BBB, supporting its use against central nervous system (CNS) malignancies [25, 26]. Indeed, 35–82% of patients with NSCLC and CNS metastases who were treated with afatinib monotherapy experienced a CNS response [27–30]. Thus, given the wider inhibitory profile of afatinib than first-generation TKIs, and its potential for CNS penetration, afatinib represents a possible treatment for GBM.

In a phase I/II study of afatinib with or without TMZ versus TMZ alone in patients with recurrent GBM, afatinib showed a manageable safety profile and modest efficacy in this hard-to-treat population [31]. There was no difference in OS between the treatment arms in the overall trial population; however, in the small number of patients assessed by biomarker subgroup analysis, there was a non-statistically significant trend towards increased PFS in afatinib-treated patients expressing the *EGFR*-variant III (*EGFR*-vIII) mutation, an *EGFR* variant frequently found in GBM [14]. Given that *EGFR* overexpression or mutation may contribute to poor outcomes and progression of GBM, and that preclinical and clinical data have highlighted potential for afatinib to elicit antitumor activity in GBM, we hypothesized that addition of afatinib to RT and TMZ may improve tumor responses and/or delay resistance to GBM treatment. The purpose of this trial was to define the toxicity and maximum tolerated dose (MTD) of afatinib in combination with RT, with and without TMZ, for the treatment of patients with newly diagnosed GBM.

## Materials and methods

### Study design and patient population

The study (NCT00977431) was a phase I, open-label, 3 + 3 dose-escalation trial in patients with newly-diagnosed malignant glioma. The trial was conducted at five sites in the United Kingdom.

Eligible patients were aged ≥ 18 and < 70 years, with newly-diagnosed, histologically-confirmed World Health Organization grade 4 malignant glioma and proven *MGMT* gene promoter methylation status (or tumor material available for testing). Exclusion criteria included: surgery within 2 weeks prior to the start of treatment or planned during the trial; placement of a Giladel^®^ wafer at surgery, prior radiotherapy of the cranium (including brachytherapy and/or radiosurgery for GBM); and treatment with other investigational drugs concomitantly with the study.

The trial was carried out in compliance with the clinical trial protocol, in accordance with the principles of the Declaration of Helsinki and International Conference on Harmonisation-Good Clinical Practice (ICH-GCP) guidelines, and in line with applicable regulatory requirements and Boehringer Ingelheim standard operating procedures. Prior to the initiation of any trial-related procedure, all patients were informed about the trial verbally and in writing by the investigator and provided written informed consent according to ICH-GCP and local legal requirements.

### Treatment

This study included two treatment regimens: regimen M, afatinib + TMZ in combination with RT; and regimen U, afatinib in combination with RT without TMZ. During the dose-finding phase, patients with methylated *MGMT* status were treated with regimen M, and patients with unmethylated *MGMT* gene promoters were treated with regimen U. The protocol was amended following the emergence of evidence demonstrating the efficacy of TMZ in patients with GBM regardless of *MGMT* methylation status [32]. Once the MTD in regimen U had been determined, all new patients were assigned to regimen M regardless of methylation status.

In both regimens, RT was administered to patients at a dose of 2 Grays (Gy) per fraction on 5 days per week for 6 weeks (total dose of 60 Gy) in an initial RT phase. Afatinib was administered in dose escalation cohorts of 20, 30, and 40 mg/day (single oral dose) during the RT phase (i.e., days 1–42), and then at 40 mg/day following RT (maintenance phase) until investigator-assessed disease progression or undue adverse reaction, whichever occurred first. For regimen M, patients received TMZ 75 mg/m^2^ daily (single oral dose) during the RT phase. A 4-week TMZ-free phase followed the RT phase, after which TMZ was administered for up to six 28-day cycles (maintenance phase: TMZ single oral dose once daily on days 1–5; 150 mg/m^2^ in cycle 1 and 200 mg/m^2^ in cycles 2–6).

Afatinib treatment was paused whenever a patient experienced an adverse event (AE) that met the criteria for dose-limiting toxicity (DLT), regardless of the cycle. DLT was defined as an AE or laboratory abnormality considered to be related to afatinib and meeting pre-specified criteria (see Supplementary Methods). Upon recovery of the AE to baseline or National Cancer Institute Common Terminology Criteria for Adverse Events (CTCAE) grade 1 (whichever was higher) within 14 days, treatment could be continued at a reduced dose. Otherwise, the patient was discontinued from trial medication, except for patients with obvious clinical benefit according to the investigator’s judgment.

### Endpoints and assessments

The primary endpoint was the MTD of continuous daily afatinib when given in combination with RT in patients with newly diagnosed GBM, with or without concomitant TMZ treatment. Secondary endpoints were the incidence and intensity of AEs, objective tumor response rate, and pharmacokinetics of afatinib (afatinib concentration at steady state, pre-dose: Days 8, 15, and 29; please see Supplemental Methods).

MTD was defined as the highest afatinib dose level at which no more than one of six patients experienced DLT, i.e., the highest afatinib dose with a DLT incidence ≤ 17%, during the 6-week RT phase. Patients who, for any reasons other than DLT, did not receive trial medication during the RT phase, for more than 5 consecutive days or more than 8 non-consecutive days, could stay in the trial, but were removed from the MTD assessment and replaced by additional patients.

Safety was assessed by physical examination, hematologic and chemistry laboratory values, vital signs, and electrocardiography scans. AEs were graded by CTCAE version 3.0. Serious AEs (SAEs) were defined as any AE that resulted in death, was immediately life threatening, resulted in persistent or significant disability, required or prolonged patient hospitalization, was a congenital anomaly/birth defect, or was deemed serious for any other reason.

Objective tumor response rate was assessed by the investigator according to the Macdonald criteria [33], as measured by cerebral gadolinium-enhanced MRI. Assessment of objective response was conducted during the maintenance phase, (i.e., following completion of radiotherapy). MRIs were performed between days 21–28 of cycles 1, 3, 5, 8, 10, and 12 for regimen M, and of cycles 2, 4, 6, 8, 10, and 12 for regimen U. In the second year, MRIs were performed every 3 months (cycles 15, 18, 21 and 24), and every 6 months thereafter. Objective response was defined as the best overall response [complete response (CR) or partial response] recorded since the first administration of treatment until disease progression, death, or treatment discontinuation. Unplanned post hoc analysis was performed to determine time to disease progression (TTP). TTP was calculated as the time between the first treatment date to the day following the first date with recorded progressive disease. Patients without progressive disease were censored at their most recent imaging date. The median and 95% confidence interval (CI) were calculated using Kaplan–Meier methodology.

### Statistical analyses

Safety, pharmacokinetic, and efficacy parameters were summarized descriptively; no formal statistical hypothesis testing was conducted. All patients who were administered at least one dose of any study treatment were included in the efficacy and safety analyses.

## Results

### Patient disposition and characteristics

Between November 2009 and October 2012, 55 patients were enrolled onto the trial. Of these, 36 patients received at least one dose of trial medication; 20 and 16 patients were treated with regimens M and U, respectively. Key baseline characteristics were similar between the two treatment arms, except for median tumor size, which was greater in patients receiving regimen U (Table 1). Patients who received regimen M were predominantly male (70%) and white (95%), with a median time from first histological diagnosis of 38 days (range 28–67) and a median tumor size (sum of largest cross-section post-surgery) of 89 mm^2^ (range 0–2088). Patients who received regimen U were also predominantly male (69%) and white (100%), with a median time from first histological diagnosis of 36 days (range 28–51) and median tumor size of 978 mm^2^ (range 0–2331).

**Table 1:** Patient baseline demographics and clinical characteristics

	Regimen M Afatinib + TMZ + RT N = 20	Regimen U Afatinib + RT N = 16
Male, n (%)	14 (70)	11 (69)
Race, n (%)		
White	19 (95)	16 (100)
Asian	1 (5)	0
Age in years, median (range)	52.5 (25–66)	53.5 (34–68)
BMI in kg/m^2^, median (range)	27.3 (20.6–33.8)	28.7 (21.7–38.8)
Smoking history, n (%)		
Never smoked	15 (75)	13 (81)
Ex-smoker	4 (20)	2 (13)
Currently smokes	1 (5)	1 (6)
Time from first histological diagnosis in days, median (range)	38.0 (28–67)	36.0 (28–51)
Karnofsky performance score, median (range)	− 10.0 (− 50–0)	− 20.0 (− 50–0)
Sum of largest cross-section post-surgery in mm^2^, median (range)	89.0 (0–2088)	978.3 (0–2331)
Unilocular, n (%)	18 (90)	15 (94)

*BMI* body mass index, *RT* radiotherapy, *TMZ* temozolomide

**Fig. 1:**
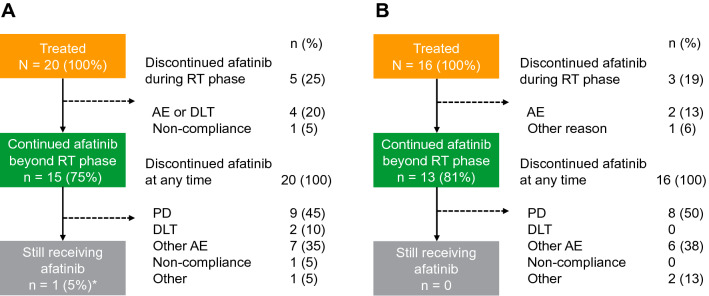
Patient disposition. **a** Regimen M: afatinib + TMZ + RT; **b** regimen U: afatinib + RT. *AE* adverse event, *DLT* dose-limiting toxicity, *PD* progressive disease, *RT* radiotherapy, *TMZ* temozolomide. *Due to trial completion, all patients are reported as having discontinued afatinib; however, one patient continued to receive afatinib outside of the clinical trial

With regimen M, 20 patients were treated and 15 patients continued afatinib beyond the RT phase. With regimen U, 16 patients were treated and 13 continued afatinib beyond the RT phase (Fig. 1). The median (range) durations of afatinib treatment for regimens M and U were 150 (6–2340) days and 167 (1–397) days, respectively.

Afatinib was discontinued by all patients during the study. Reasons for afatinib discontinuation with regimen M included disease progression (45%), DLT (10%), and other AEs (35%) (Fig. 1). One patient who received regimen M had not experienced disease progression at data cut-off and was switched to commercially supplied afatinib; he remained on treatment for more than 6 years. With regimen U, reasons for afatinib discontinuation included disease progression (50%), and AEs other than DLTs (38%); no patients receiving regimen U discontinued afatinib treatment due to DLTs.

### MTD of afatinib with concomitant RT

**Fig. 2:**
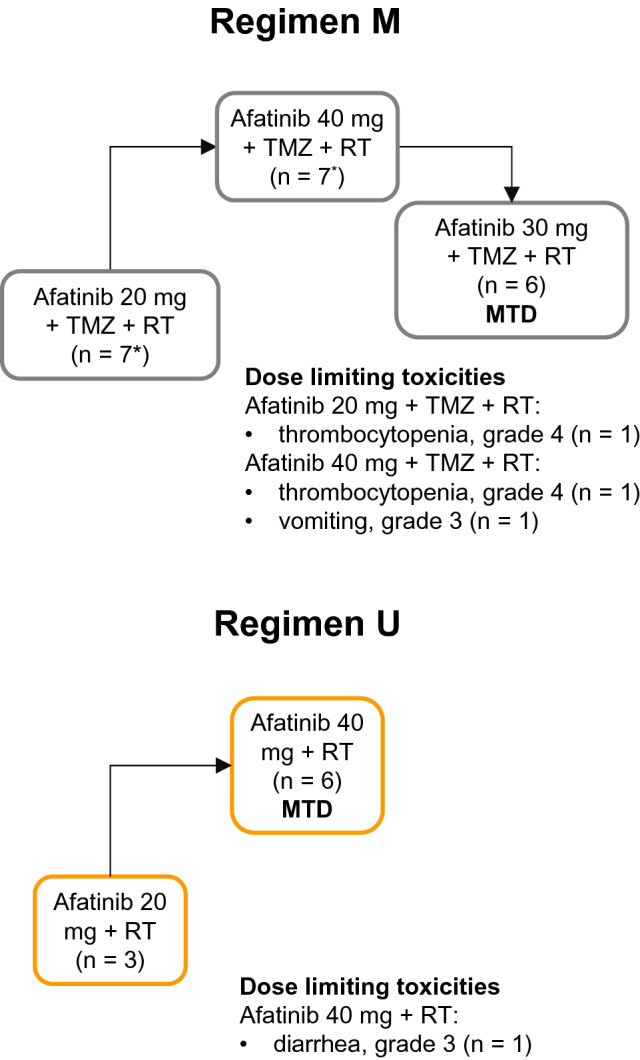
Determination of the maximum tolerated dose based on the occurrence of dose limiting toxicities during the 6-week radiotherapy phase. *MTD* maximum tolerated dose, *RT* radiotherapy, *TMZ* temozolomide. *In Regimen M, one patient was replaced in the 20 mg afatinib group and one patient was replaced in the 40 mg afatinib group

Overall, 17 of 20 patients who received regimen M were evaluable for MTD determination (Fig. 2). In the first dose cohort (afatinib 20 mg/day), one of six evaluable patients had a DLT during the RT phase (grade 4 thrombocytopenia). The afatinib dose was therefore escalated to 40 mg/day; two of five patients (seven patients were treated; two were not evaluable) had DLTs: one patient had grade 4 thrombocytopenia and one patient had grade 3 vomiting. The afatinib dose was reduced and an intermediate dose level of 30 mg/day was explored; none of the six treated patients had DLTs. Accordingly, afatinib 30 mg was determined as the MTD in combination with daily TMZ and RT.

With regimen U, nine of 16 patients treated were evaluable for MTD determination. In the first dose cohort (afatinib 20 mg/day), three patients were treated without any DLT during the RT phase. The afatinib dose was subsequently escalated to 40 mg/day; one of six patients had a DLT (grade 3 diarrhea). Thus, afatinib 40 mg was determined as the MTD in combination with RT.

For pharmacokinetic data, please see Supplemental Results.

### Safety profile of each regimen

The most frequently reported AEs with regimen M were diarrhea (85%), nausea (75%), and rash (65%). Of patients receiving regimen M, 95% had at least one drug-related AE (Table 2); the most common drug-related AEs were diarrhea (80%), rash (65%), nausea (45%), and fatigue (45%; Supplementary Table 1). Nine (45%) patients had AEs that led to discontinuation of afatinib; AEs (preferred terms) reported in more than one patient were diarrhea, fatigue, rash, and thrombocytopenia (two patients each). All other AEs leading to afatinib discontinuation were reported in single patients only; these were skin toxicity, skin ulcer, alanine aminotransferase increased, and postoperative wound infection.

**Table 2:** Summary of AEs

	Regimen M Afatinib (20 mg) + TMZ + RT N = 7	Regimen M Afatinib (30 mg) + TMZ + RT N = 6	Regimen M Afatinib (40 mg) + TMZ + RT N = 7	Regimen M Afatinib (total) + TMZ + RT N = 20
Any AE, n (%)	7 (100)	6 (100)	7 (100)	20 (100)
DLTs^a^, n (%)	4 (57)	1 (17)	3 (43)	8 (40)
Treatment-related AEs, n (%)	7 (100)	6 (100)	6 (86)	19 (95)
AEs leading to discontinuation of afatinib, n (%)	4 (57)	0	5 (71)	9 (45)
AEs leading to discontinuation of TMZ, n (%)	1 (14)	0	2 (29)	3 (15)
SAEs^b^, n (%)	4 (57)	5 (83)	3 (43)	12 (60)
Maximum CTCAE grade, n (%)				
Grade 1	0	0	1 (14)	1 (5)
Grade 2	0	2 (33)	1 (14)	3 (15)
Grade 3	4 (57)	4 (67)	4 (57)	12 (60)
Grade 4	3 (43)	0	1 (14)	4 (20)
Grade 5	0	0	0	0

	Regimen U afatinib (20 mg) + RT N = 3	Regimen U afatinib (40 mg) + RT N = 13	Regimen U afatinib (total) + RT N = 16
Any AE, n (%)	3 (100)	13 (100)	16 (100)
DLTs^a^, n (%)	2 (67)	4 (31)	6 (38)
Treatment-related AEs, n (%)	3 (100)	12 (92)	15 (94)
AEs, regardless of causality, leading to discontinuation of afatinib, n (%)	1 (33)	9 (69)	10 (63)
SAEs^b^, n (%)	2 (67)	10 (77)	12 (75)
Maximum CTCAE grade, n (%)			
Grade 1	0	1 (8)	1 (6)
Grade 2	1 (33)	2 (15)	3 (19)
Grade 3	2 (67)	5 (38)	7 (44)
Grade 4	0	2 (15)	2 (13)
Grade 5	0	3 (23)	3 (19)

*AE* adverse event, *CTCAE* Common Terminology Criteria for Adverse Events, *DLT* dose-limiting toxicity, *RT* radiotherapy, *SAE* serious adverse event, *TMZ* temozolomide

^a^DLTs occurring in the overall treatment period, as determined by the investigator

^b^A patient may have experienced more than one SAE

With regimen U, the most frequently reported AEs were diarrhea (94%), rash (75%), and headache (63%). Of patients receiving regimen U, 94% of patients had at least one drug-related AE (Table 2); the most frequently reported drug-related AEs were diarrhea (81%), rash (75%), and fatigue (45%; Supplementary Table 1). Ten patients (63%) had AEs that led to discontinuation of afatinib; these were diarrhea, dermatitis acneiform, rash, pneumonia, pulmonary embolism, generalized tonic–clonic seizure, increased intracranial pressure, lethargy, malignant neoplasm progression, and disease progression (one patient each).

Frequencies of grade ≥ 3 drug-related AEs and SAEs by treatment regimen and dose are shown in Table 2. SAEs were reported in 12 (60%) patients who received regimen M and 12 (75%) patients who received regimen U. There were no fatal AEs reported for patients who received regimen M. Fatal AEs were reported in three patients (19%) who received regimen U. The causes of death in these patients were bacterial meningitis, pneumonia, and disease progression. For all three deaths, none were considered to be drug-related.

### Response to therapy

Of 20 evaluable patients who received regimen M, five (25%) patients achieved an objective response, including one CR, and 11 (55%) patients had stable disease. With regimen U, one (6%) of 16 evaluable patients had an objective response and eight (50%) had stable disease according to the Macdonald response assessment criteria [33]. The best overall responses by afatinib dose with regimens M and U are shown in Table 3. The median time to disease progression for evaluable patients who received regimens M and U were 434 days (95% CI 205–NA, n = 18) and 211 days (95% CI 101–NA, n = 14), respectively. Insufficient data were available to calculate upper 95% CIs.

**Table 3:** Summary of treatment response

	Regimen M Afatinib (20 mg) + TMZ + RT N = 7	Regimen M Afatinib (30 mg) + TMZ + RT N = 6	Regimen M Afatinib (40 mg) + TMZ + RT N = 7	Regimen M Afatinib (total) + TMZ + RT N = 20
Best overall response, n (%)				
CR	0	1 (17)	0	1 (5)
PR	2 (29)	2 (33)	0	4 (20)
SD	5 (71)	2 (33)	4 (57)	11 (55)
PD	0	1 (17)	1 (14)	2 (10)
Missing	0	0	2 (29)	2 (10)
Objective response, n (%)	2 (29)	3 (50)	0	5 (25)
Disease control rate, n (%)	7 (100)	5 (83)	4 (57)	16 (80)

	Regimen U afatinib (20 mg) + RT N = 3	Regimen U afatinib (40 mg) + RT N = 13	Regimen U afatinib (total) + RT N = 16
Best overall response, n (%)			
CR	0	0	0
PR	0	1 (8)	1 (6)
SD	1 (33)	7 (54)	8 (50)
PR	2 (67)	3 (23)	5 (31)
Missing	0	2 (15)	2 (13)
Objective response, n (%)	0	1 (8)	1 (6)
Disease control rate, n (%)	1 (33)	8 (62)	9 (56)

*CR* complete response, *PD* progressive disease, *PR* partial response, *RT* radiotherapy, *SD* stable disease, *TMZ* temozolomide

## Discussion

In this open-label, phase I dose-escalation trial in newly diagnosed patients with GBM, the MTD of afatinib was 30 mg/day in combination with RT and TMZ (regimen M; methylated *MGMT* promoter), and 40 mg/day in combination with RT (regimen U; unmethylated *MGMT* promoter).

The most frequently reported drug-related AEs in this trial with regimens M/U were diarrhea (80/81%), rash (65/75%), and fatigue (45/38%), with nausea also reported in a high proportion of the patients who received regimen M (45%). These findings are similar to the known toxicity profile of afatinib when used as a single agent in patients with NSCLC [28, 34], and also to those observed in the phase I/II study of afatinib with or without TMZ in recurrent GBM, in which the most frequent AEs observed in afatinib-containing arms (afatinib alone/afatinib + TMZ) were rash/acne and diarrhea [31]. The AE profiles of regimens M and U were also consistent with those of the afatinib combination partners, and, aside from grade 4 thrombocytopenia reported in three patients (15%) receiving the TMZ-containing regimen M, there was no evidence that afatinib increased the incidence of TMZ-associated toxicities, compared with previous reports of TMZ with or without afatinib [5, 31].

The pharmacokinetics of afatinib in combination with RT, with or without TMZ, appeared to be consistent with those previously reported for single-agent afatinib [35]. There were no meaningful differences in afatinib trough plasma concentrations over time (Days 8, 15, and 29 from start of treatment) nor between treatment regimens, suggesting that concentrations were unaffected by RT or TMZ.

The key aim of this study was not to investigate efficacy; however, disease control observed at RT completion was indicative of modest efficacy for both treatment regimens in patients with newly diagnosed GBM. It is unclear whether treatment with afatinib was a contributing factor, as it was administered in combination with treatments with known efficacy in GBM [5, 32]. Moreover, previous studies of ErbB pathways inhibitors in GBM have shown little efficacy when given alone [31, 36]. In a phase Ib/II trial of afatinib with or without TMZ in patients with recurrent GBM, the 6-month PFS rate was significantly lower with afatinib monotherapy than with afatinib plus TMZ or TMZ alone (afatinib alone: 3%; afatinib + TMZ: 10%; TMZ alone: 23%) [31]. However, median PFS was longer in afatinib-treated patients with EGFR*-*overexpressing tumors (3.35 months) than those with EGFR levels within a normal range (0.99 months). Similar results have been observed with other EGFR TKIs. Gefitinib (with or without chemotherapy) was associated with response rates of up to 14% and 6-month PFS rates of 5–24% in patients with recurrent glioma [36–39]. Similarly, response rates of up to 8% have been achieved with erlotinib, with little impact on overall response or PFS in patients with recurrent malignant glioma compared with TMZ (with or without chemotherapy) [40–43]. While previous studies have indicated minimal activity of ErbB family inhibitors in GBM, a case of prolonged response to afatinib has been reported previously in a patient with recurrent GBM in this study. This patient, who had several *EGFR* mutations, *EGFR* gene amplification, and *EGFR*-vIII seropositivity, survived for around 5 years from recurrence, nearly sixfold longer than expected in patients with recurrent GBM [44, 45]. The patient was switched to commercial supply and was still on treatment at the time of the database lock. A further two patients with GBM who had long-term responses (> 12 months) to afatinib harbored mutations in specific combinations of alleles that are causal of EGFR addiction [44, 46]. For example, one patient had a *PTPN11* mutation thought to drive EGFR addiction and, hence, response to afatinib, and another patient had a tumor that was *EGFR* amplified and carried an additional allele on the amplicon, potentially underlying the sustained response observed [46]. These findings suggest that afatinib may be of most benefit in patients with GBM harboring *EGFR* aberrations.

Given that alterations affecting EGFR*,* e.g. EGFR overexpression, have been identified previously in tumors of patients with GBM, including in long-term responders to afatinib [16, 17, 44, 46], a potential limitation of the present study is that patients were not selected based on biomarker analysis. Patients were not selected in this manner as *EGFR* genetic testing was not routinely performed when the trial was initiated. In future trials, selection of patients based upon specific biomarkers, such as *EGFR* mutations or amplification, may assist in identifying patients who are more likely to benefit from EGFR-targeted therapies. Another limitation of this study is that, similar to other trials to date, it has not been possible to distinguish the efficacy of afatinib from the known effectiveness of RT and TMZ [31]. Additionally, response to therapy was evaluated using Macdonald criteria, which were in widespread use at the time of the design of this study. These criteria have largely been superseded by response assessment in neuro-oncology (RANO) criteria [47, 48].

This dose-finding study identified the MTD for afatinib in combination with RT and TMZ for patients with methylation of the *MGMT* promoter (30 mg/day during RT; 40 mg/day maintenance phase; regimen M), and in combination with RT for patients without methylation of the *MGMT* promoter (40 mg/day in RT and maintenance phases; regimen U). Treatment with both regimens was associated with a manageable AE profile that was consistent with the known safety profiles of the individual agents; the pharmacokinetic profile of afatinib was also in line with previous afatinib monotherapy studies at all dose levels. While this study only included a small number of patients, and efficacy was not the primary endpoint, antitumor activity was observed in a subset of each treatment group. Given the relationship between *EGFR* aberrations and poor response to treatment in GBM, the ErbB pathway remains a plausible therapeutic target in GBM. Research into the safety and pharmacokinetics of afatinib in patients with previously treated brain cancer is ongoing in a phase I study (NCT02423525). In future studies, biomarker analysis should be utilized to guide preselection of patients most likely to benefit from afatinib treatment.

## Supplementary Information

Below is the link to the electronic supplementary material.

Supplementary file1 (DOCX 78 kb)

## Data Availability

To ensure independent interpretation of clinical study results, Boehringer Ingelheim grants all external authors access to relevant material, including participant-level clinical study data, as needed by them to fulfill their role and obligations as authors under the ICMJE criteria. Clinical study documents and participant clinical study data are available to be shared on request after publication of the primary manuscript in a peer-reviewed journal, and if regulatory activities are complete and other criteria met as per the BI Policy on Transparency and Publication of Clinical Study Data (see https://www.mystudywindow.com/msw/datasharing). Bona fide, qualified scientific and medical researchers are eligible to request access to the clinical study data with corresponding documentation describing the structure and content of the datasets. Upon approval, and governed by a Legal Agreement, data are shared in a secured data-access system for a limited period of 1 year, which may be extended upon request. Prior to providing access, clinical study documents and data will be examined, and, if necessary, redacted and de-identified, to protect the personal data of study participants and personnel, and to respect the boundaries of the informed consent of the study participants. Researchers should use the https://vivli.org/ link to request access to study data and visit https://www.mystudywindow.com/msw/datasharing for further information.

## Code availability

(software application or custom code): not applicable.
