# Supplementary material for: Afatinib and radiotherapy, with or without temozolomide, in patients with newly diagnosed glioblastoma: results of a phase I trial
Source: J Neurooncol. 2021 Nov 17;155(3):307–17. doi: 10.1007/s11060-021-03877-6 (PMC8651574; doi:10.1007/s11060-021-03877-6)
Supplement: Supplementary file 1 — Supplementary file1 (DOCX 78 kb) [file 11060_2021_3877_MOESM1_ESM.docx]

**Supplementary Material**

**Supplementary methods**

**Definition of dose-limiting toxicity**

A dose-limiting toxicity (DLT) was defined as an adverse event (AE) or laboratory abnormality considered related to afatinib and meeting any of the following criteria:

1. Hematologic adverse reaction:
2. Common Terminology Criteria for Adverse Events (CTCAE) grade 4 neutropenia (absolute neutrophil count, including bands, < 500/mm³) for > 7 days
3. CTCAE grade 3 or 4 neutropenia of any duration associated with fever >38.3°C
4. CTCAE grade 3 thrombocytopenia (platelet count < 50,000–25,000/mm³)
5. All other hematologic toxicities of CTCAE grade ≥ 3 leading to an interruption of afatinib treatment (regimen U) or treatment with afatinib and TMZ (regimen M) for > 14 days
6. Non-hematologic adverse reaction:
7. CTCAE grade ≥ 3 nausea or vomiting despite appropriate use of standard anti-emetics for ≥ 3 days
8. CTCAE grade ≥ 3 diarrhea despite appropriate use of standard anti-diarrheal therapy for ≥ 3 days
9. CTCAE grade ≥ 3 rash despite standard medical management and lasting > 7 days
10. CTCAE grade ≥ 2 cardiac left ventricular function
11. CTCAE grade ≥ 2 worsening of renal function as measured by serum creatinine, newly developed proteinuria, or newly developed decrease in glomerular filtration rate
12. All other non-hematologic toxicities of CTCAE grade ≥ 3

**Pharmacokinetic sampling**

Samples were taken pre-dose on days 8, 15, and 29 of treatment, as well as 1, 3, and 6 hours post-dose on day 15 (the 6-hour post-dose sample was voluntary). Post-dose plasma drug concentrations are not further discussed.

**Supplementary results**

**Pharmacokinetics of afatinib**

With regimen M, two patients had measurements available for afatinib trough plasma concentrations at all three time points with 40 mg afatinib. Four additional patients had measurements for at least one time point. With regimen U, nine patients had measurements available for afatinib trough plasma concentrations at all three time points with 40 mg afatinib. In addition, 40 mg data were available for at least one time point from three additional patients. Afatinib trough plasma concentrations appeared stable throughout the treatment period and were similar between treatment regimens. Steady-state geometric mean plasma concentrations of afatinib with regimen M (40 mg/day) for days 8, 15, and 29 were 15.7−17.4 ng/mL (Supplementary Figure 1). For regimen U (40 mg/day), the concentrations were 16.1−18.9 ng/mL.

**Supplementary Figure 1** Geometric mean afatinib pre-dose steady-state plasma concentrations. (**a**) Regimen M: afatinib+TMZ+RT; (**b**) Regimen U: afatinib+RT. *gMean* geometric mean, *RT* radiotherapy, *TMZ* temozolomide.


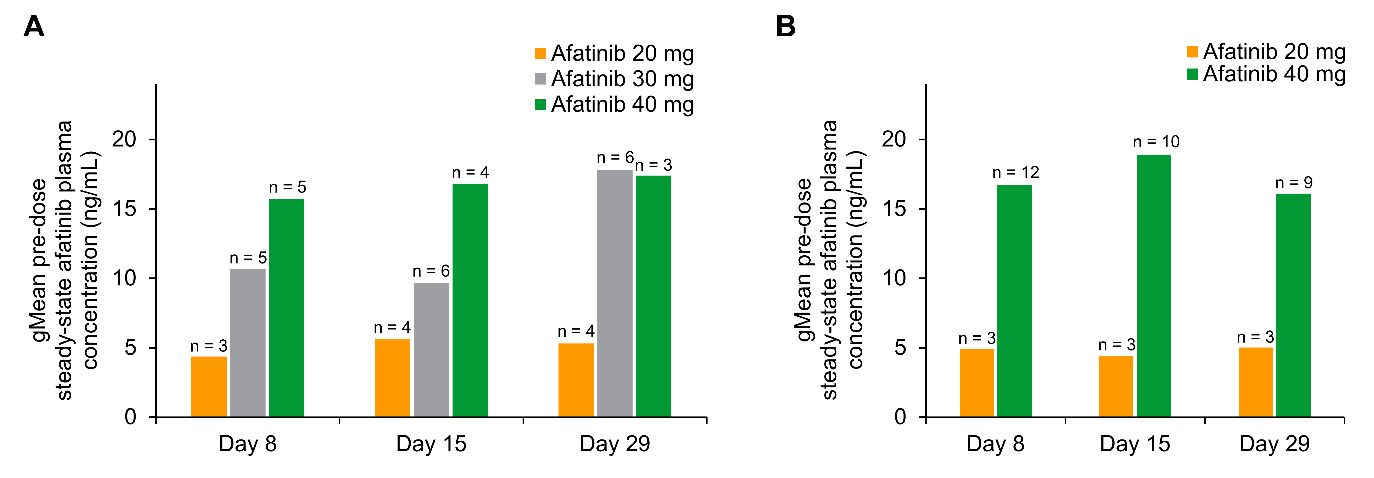


**Supplementary Table 1** Drug-related AEs in ≥ 20% of patients overall by primary SOC and preferred term

|  | **Regimen M  Afatinib (20 mg) +TMZ+RT**  **N = 7** | **Regimen M  Afatinib (30 mg) +TMZ+RT**  **N = 6** | | **Regimen M  Afatinib (40 mg) +TMZ+RT**  **N = 7** | | **Regimen M  Afatinib (total) +TMZ+RT**  **N = 20** |
| --- | --- | --- | --- | --- | --- | --- |
| Total with related AE, n (%) | 7 (100) | 6 (100) | | 6 (86) | | 19 (95) |
| Gastrointestinal disorders | 6 (86) | 6 (100) | | 5 (71) | | 17 (85) |
| Diarrhea | 6 (86) | 5 (83) | | 5 (71) | | 16 (80) |
| Nausea | 3 (43) | 2 (33) | | 4 (57) | | 9 (45) |
| Skin and subcutaneous tissue disorders | 6 (86) | 5 (83) | | 5 (71) | | 16 (80) |
| Rash | 5 (71) | 5 (83) | | 3 (43) | | 13 (65) |
| General disorders and administration site conditions | 6 (86) | 4 (67) | | 2 (29) | | 12 (60) |
| Fatigue | 4 (57) | 3 (50) | | 2 (29) | | 9 (45) |
| Infections and infestations | 2 (29) | 2 (33) | | 2 (29) | | 6 (30) |
| Blood and lymphatic system disorders | 3 (43) | 1 (17) | | 2 (29) | | 6 (30) |
|  | **Regimen U  afatinib (20 mg)**  **+RT**  **N = 3** | | **Regimen U afatinib (40 mg)**  **+RT**  **N = 13** | | **Regimen U  afatinib (total)**  **+RT**  **N = 16** | |
| Total with related AE, n (%) | 3 (100) | | 12 (92) | | 15 (94) | |
| Skin and subcutaneous tissue disorders | 3 (100) | | 11 (85) | | 14 (88) | |
| Rash | 2 (67) | | 10 (77) | | 12 (75) | |
| Gastrointestinal disorders | 3 (100) | | 10 (77) | | 13 (81) | |
| Diarrhea | 3 (100) | | 10 (77) | | 13 (81) | |
| General disorders and administration site conditions | 3 (100) | | 3 (23) | | 6 (38) | |
| Fatigue | 3 (100) | | 3 (23) | | 6 (38) | |
| Infections and infestations | 1 (33) | | 3 (23) | | 4 (25) | |
| Nervous system disorders | 1 (33) | | 3 (23) | | 4 (25) | |

*AE* adverse event, *RT* radiotherapy, *SOC* system organ class, *TMZ* temozolomide
